# The Relationship of Immune Cell Signatures to Patient Survival Varies within and between Tumor Types

**DOI:** 10.1371/journal.pone.0138726

**Published:** 2015-09-23

**Authors:** Peter S. Linsley, Damien Chaussabel, Cate Speake

**Affiliations:** Department of Systems Immunology, Benaroya Research Institute, 1201 Ninth Avenue, Seattle, WA 98101–2795, United States of America; Princess Margaret Cancer Centre, CANADA

## Abstract

Enhancing pre-existing anti-tumor immunity leads to therapeutic benefit for some patients, but why some tumors are more immunogenic than others remains unresolved. We took a unique systems approach to relate patient survival to immune gene expression in >3,500 tumor RNAseq profiles from a dozen tumor types. We found significant links between immune gene expression and patient survival in 8/12 tumor types, with tumors partitioned by gene expression comprising distinct molecular subtypes. T/NK cell genes were most clearly survival-related for melanoma, head and neck, and bladder tumors, whereas myeloid cell genes were most clearly survival-related with kidney and breast tumors. T/NK or myeloid cell gene expression was linked to poor prognosis in bladder and kidney tumors, respectively, suggesting tumor-specific immunosuppressive checkpoints. Our results suggest new biomarkers for existing cancer immunotherapies and identify targets for new immunotherapies.

## Introduction

Recent advances in immunotherapy of cancer have led to durable clinical responses to multiple immunotherapeutic agents across a range of human cancers [1,2]. One particularly promising therapeutic approach, termed 'checkpoint blockade' [2,3], utilizes monoclonal antibodies (mAbs) that block immune-inhibitory pathways switched on by cancer cells. Despite advances, cancer immunotherapy is not always effective and may be associated with significant safety issues [4,5], leading to intensive efforts to identify biomarkers for better patient selection [6,7]. It remains unclear why some tumors respond to immunotherapy and why others do not (i.e., why some tumors are more “immunogenic” than others).

One predictor of cancer patient prognosis is the presence and location of immune infiltrates within tumors. Immunohistochemical studies have shown that immune cell infiltrates in tumors at diagnosis can be linked to favorable clinical outcome [8,9]. Expression profiling has also provided evidence of immune cell infiltrates and their relationship to patient survival in cancer. Comparisons of multiple different molecular profiling studies have suggested common themes in immune cell infiltrates across different tumor types reviewed in [10,11]. More recent studies have suggested that a therapeutic response to anti-PD-1 monoclonal antibody (mAb) requires pre-existing PD-1/PD-L1-regulated T cells within tumors [6,7,12].

Despite abundant evidence linking tumor immune infiltrates with patient prognosis and response to therapy, most studies have focused on single tumor types and comparisons across different tumors largely have been based on literature comparisons [10]. Resolving the uncertainties and gaps in our knowledge would benefit from a direct, side-by-side comparison of immune mechanisms influencing patient survival across different tumor types. The Cancer Genome Atlas (TCGA) is a comprehensive effort to apply genome analysis technologies to accelerate understanding of the molecular basis of cancer [13]. In particular, the Pan-Cancer initiative involving the first 12 tumor types profiled by TCGA has been used to identify commonalities and differences across tumor lineages, including survival comparisons of patients with tumors of different molecular subtypes [13,14] We reasoned that TCGA RNAseq data from the Pan-Cancer initiative could be used for side-by-side testing to identify immune signatures linked to patient survival, both within and between different tumor types.

We recently described a novel approach, termed module analysis, to analyze melanoma RNA sequencing expression data (RNAseq) for immune cells and pathways linked to patient survival [15]. Our studies showed that levels of type I interferon-stimulated genes (ISGs), and T cell genes in melanomas at the time of diagnosis significantly predicted patient survival. In the present study, we have used expanded modular gene expression analysis on combined data from the TCGA Pan-Cancer and melanoma profiling initiatives. We present for the first time a comparison of immune cells and pathways associated with patient survival across a dozen different tumor types. The results provide a richer look at immune cell infiltrates and patient survival than has been possible with previous studies focused on individual tumor types.

## Results

### Different immune processes are associated with patient survival after tumor detection

We analyzed a combined set of >3,500 RNAseq profiles from the TCGA Pan-Cancer and melanoma profiling initiatives (Experimental Procedures). Tumor biopsies were taken near the time of diagnosis. The tumor types and numbers of profiles involved are described in Table 1, along with abbreviations used for tumor types.

**Table 1:** Characteristics of tumors examined in this study.

tumor abbreviation	full name	TCGA code	number profiles	median survival (days)
Bladder	Bladder Urothelial Carcinoma	BLCA	84	219
Breast	Breast invasive carcinoma	BRCA	748	578
Colon	Colon adenocarcinoma	COAD	108	376
Gliobastoma	Glioblastoma multiforme	GBM	159	285
Head and Neck	Head and Neck squamous cell carcinoma	HNSC	299	450
Kidney	Kidney renal clear cell carcinoma	KIRC	425	1097
Lung adenocarcinoma	Lung adenocarcinoma	LUAD	288	316
Lung squamous cell	Lung squamous cell carcinoma	LUSC	204	576
Ovarian	Ovarian serous cystadenocarcinoma	OV	261	882
Rectal	Rectum adenocarcinoma	READ	42	329
Melanoma	Skin Cutaneous Melanoma	SKCM	274	1345
Uterine	Uterine Corpus Endometrial Carcinoma	UCEC	332	553

We used a strategy described previously [15] to query these tumor datasets for the relationship between levels of immune gene expression and patient survival. Briefly, this involved testing whether levels of immune gene sets (modules) in tumors could predict patient survival. To minimize biases, we chose not to aggregate clinical data from disparate tumor types, but instead analyzed each tumor dataset separately, using the procedures that we validated in our previous studies [15]. For the current studies, we developed a custom set of transcript modules whose expression was most associated with selected marker genes (immune molecular modules, S1 and S2 Tables).

We chose to use custom modules rather than previously described transcript modules [16–18] for several reasons. A major reason was that previously described transcript modules vary widely in the numbers of genes they comprise [16–18]. This introduces a complicating variable (set size differences) into survival comparisons of tumor subsets. Using custom modules of equivalent set size mitigates this problem. The use of custom modules also allowed us to preselect a set of markers to use as queries that are not well represented in previous module sets. Finally, our custom modules can be organized into groups or clusters by their degree of gene overlap (Experimental Procedures and [15]). These clusters of overlapping modules permit greater insight into the scope and depth of transcriptional programs in immune cell infiltrates, as well as specificity control. Our approach was validated by our previous demonstration of a strong ISG response in melanomas [15]. For the present studies, we used a more inclusive set of marker genes and a larger data set to identify modules.

In preliminary experiments, we compared survival of tumor subsets of varying size. In other experiments, we determined optimal grouping for survival comparisons by subjecting samples to hierarchical clustering according to module expression. We used this method previously to show that ISG expression levels were dose-dependent in their ability to predict melanoma patient survival [15]. In general, we found consistent results using sample subsets of different sizes, which justified the selection of an arbitrary standard for sample set size. For the experiments reported here, we used expression of module genes to partition each of the tumor data sets (module partitions) into subsets with greater or less than median expression of module genes (designated module hi and module lo subsets, respectively) [15].

We then compared survival curves of the two patient subsets using a log-rank test [19]. We used this procedure to test all immune molecular modules (N = 526) for their ability to predict survival in each of 12 tumor types (S3 Table). Also shown in S3 Table are the prognostic values of the immune molecular module signatures for different tumor types, as estimated using an implementation [19] of the Concordance index [20,21]. The Concordance index is a generalization of an area under the ROC curve measurement that indicates how well a model discriminates between responses. A Concordance index value = 0.5 implies no predictive ability, whereas values > 0.5–1.0 imply positive predictive ability. The mean Concordance index value was 0.59 for immune molecular module signatures passing a stringent p-value cutoff of <1e-3, indicating positive predictive value.

**Fig 1:**
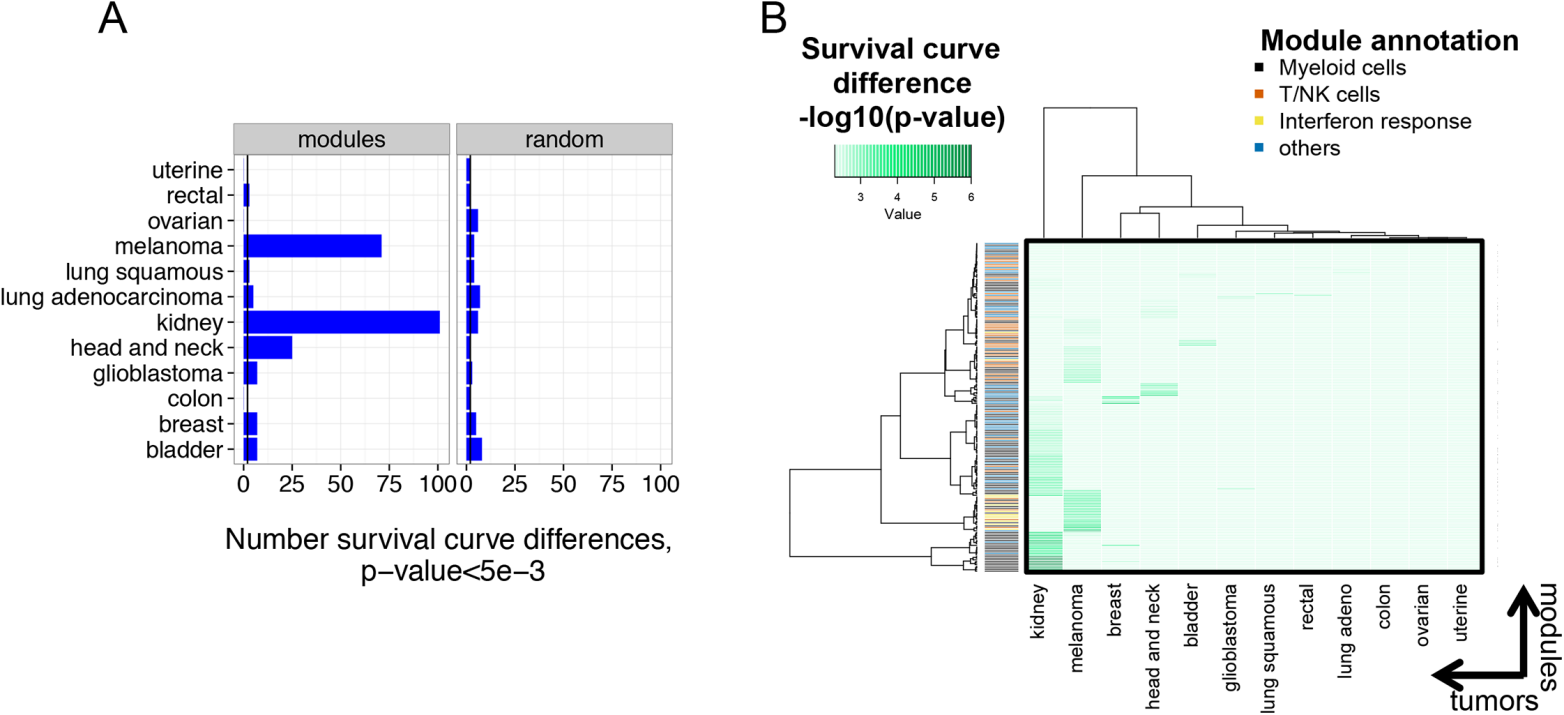
Immune gene expression in tumors predicts patient survival in some but not all tumor types. A) Numbers of immune molecular modules predicting survival varies between tumor types. Tumors were partitioned into equal sized groups by median gene expression of each of 526 immune molecular modules (Experimental Procedures). Shown are numbers of modules that significantly predicted survival of patients with each of twelve different tumor types (p-value<5e-3). The numbers of random partitions that yielded significant survival curve differences for each tumor are shown for comparison (N = 526 permutations). At this p-value, ~2 partitions would be expected to yield significant survival curve differences by chance (vertical lines). B) Qualitative differences in the spectrum of modules yielding significant survival curve differences in different tumors. The heatmap displays different tumor types on the x axis versus the identities of molecular modules that gave significant survival curve differences in any tumor type at p-value <5e-3 (N = 229) on the y axis (S3 Table). The green color intensity indicates the significance of differences in survival curves (-log10 (p-value)). The color bar indicates the summary annotation term for each module on the y axis. Clustering was performed in both the x and y dimensions.

To control false discovery rates, we randomly used random partitions of both samples and genes. As described below, we considered random sample partitions to more accurately describe the null distribution for comparison of gene sets. We partitioned samples from each tumor type into equal sized groups (random partitions). From 1,000 random partitions of each tumor samples into equally sized groups, we observed an average of ~1 partition for each tumor type that predicted patient survival at *survdiff* p-values <1e-3, and ~4 modules at p-values <5e-3. For partitions by immune molecular module (N = 526), we therefore expected <~1 and ~2 survival curve differences by chance at p-values <1e-3, and <5e-3, respectively. We then compared the number of times that module and random partitions predicted significant differences in survival for each tumor type. Graphical comparisons of results from module and random partitions at p-value <5e-3 are shown in S1 Fig and Fig 1A.

We found numerous significant patient survival differences between module hi and module lo subsets in certain tumor types (S1 Fig and Fig 1A). Few random partitions yielded significant survival curve differences in any tumor type (S1 Fig). The highest numbers of significant survival differences were in kidney, melanoma, head and neck, bladder, and breast tumors. Strikingly, kidney, melanoma, head and neck, and bladder tumors are among the tumor types most treatable with therapies that depend upon engagement of the host immune system (i.e., immune-based therapies) [22]. Module partitions yielded fewer significant survival differences in patients with glioblastoma, colon, uterine, rectal, lung squamous, lung adenocarcinoma, and ovarian tumors, tumors that generally are not treatable with immune-based therapy.

It was important to exclude trivial technical factors as an explanation for the significant survival differences seen with module partitions. The number of modules significantly associated with survival differences was not clearly related to numbers of tumor samples analyzed. For instance, breast and bladder carcinomas showed similar survival differences, despite great variation in the numbers of tumor samples tested (Fig 1A and Table 1). It is well known that prognostic value of gene signatures depends on the length of follow-up [23]. Since the survival time varies for the different tumors in the PANCANCER set (Table 1), we were concerned about the possibility that differences in follow-up time could have biased our results.

Examination of our data did not reveal a clear relationship to median survival of different tumor types. For instance, both bladder and glioblastoma tumors were associated with the shortest median survival times (both <300 days) but only bladder carcinoma showed clear survival differences with module partitions. Moreover, ovarian tumors had among the highest median survival times (882 days), but did not show any modules associated with significant survival differences. To examine the effects of different follow-up times more directly, we attenuated the differences in follow-up length by truncated the data to a common, somewhat short follow-up length (<5 years). The truncated data set showed a loss of power expected for reduced sample numbers, but very similar overall trends in survival differences as the full data set (S3 Table). Taken together, these findings suggest that differences in follow-up time did not greatly affect our overall results.

We further investigated the role of technical factors by determining whether the same or different biological processes were associated with modules showing significant survival differences (Fig 1A). Technical factors affecting the ability to detect survival differences in certain tumors might be expected to result in similar immune processes linked to survival across multiple tumors. Alternatively, qualitatively different immune processes related to patient survival in different tumors would argue that survival differences are biologically based. To distinguish these possibilities, we performed a global comparison of survival-associated modules across all tumor types. We selected modules that were associated with significant survival differences in any tumor type (S3 Table, p-value <5e-3) and compared their performance across all other tumor types (Fig 1B). This analysis showed qualitatively distinct groups of modules most associated with survival differences in each tumor type. For instance, modules associated with Myeloid cells were most highly associated with survival of kidney tumor patients, whereas Interferon response and T/NK cell modules were most effective with melanoma [15]. Breast, head and neck and bladder tumors also showed distinct patterns, but the remaining tumor types did not clearly show clusters of modules yielding significant survival advantage, consistent with Fig 1A.

To gain a more in depth view of immune processes associated with patient survival, we parsed the complete data set (S3 Table) for processes represented by modules associated with survival differences. Using both stringent and less-stringent p-value cuts (1e-3 and 5e-3, respectively), we determined numbers of modules yielding survival differences for each tumor type (Table 2). In order to reduce the impact of tallying modules comprised of very similar genes (a form of double counting), we also determined the numbers of clusters represented by significant modules. For comparison, we also present the numbers of random sample and gene set partitions that yielded significant survival differences. In addition we mined the annotation terms representing each cluster to draw biological inferences. To minimize false positive detections, we focused only cases where we saw significant survival differences with multiple modules from the same cluster, or with modules from different clusters having similar biological annotation.

**Table 2:** Distinct functional patterns in modules predicting survival in different tumor types.

Tumor	No. random samples	No. random gene sets	No. modules	No. clusters	Selected top modules	Top modules annotation
Kidney	**0**, 1	**18**, 41	**56**, 101	11, 14	**INSL3.mod**, **CTSG.mod, BST2.mod, E2F1.mod**	**Myeloid cells, Myeloid cells, Interferon response, T/NK cells**
Melanoma	**1**, 2	**4**, 14	**33**, 71	**10**, 16	**STAT2.mod, SEMA3B.mod, FLT3LG.mod**	**Interferon response, Myeloid cells, T/NK cells**
Head & neck	**1**, 6	**2**, 16	**6,** 25	**4,** 9	**TNFRSF9.mod**, PDCD1.mod	**Myeloid cells,** T/NK cells
Bladder	**0**, 5	**1**, 9	**3**, 7	**2**, 4	**FOXP3.mod,** ALCAM.mod	**T/NK cells,** Myeloid cells
Breast	**0**, 3	**8**, 20	**5**, 7	**3**, 4	CD63.mod, DEFA3.mod	Myeloid cells, Myeloid cells
Lung adeno.	**2**, 3	**0**, 6	**0,5**	**0,** 4	CD19.mod	B cells
Glioblastoma	**0**, 1	**4**, 11	**0,** 7	**0,** 6	NA	NA
Colon	**1**, 3	**1**, 4	**0**,0	**0**,0	NA	NA
Uterine	**0**, 1	**0**, 3	**0**,0	**0**,0	NA	NA
Rectal	**0**, 0	**0**, 0	**0**,3	**0**,2	NA	NA
Lung sq.	**0**, 4	**0**, 1	**0**,3	**0**,3	NA	NA
Ovarian	**0**, 3	**2**, 5	**0**,0	**0**,0	NA	NA

Shown is a summary of properties of immune molecular modules whose expression in tumors significantly predicts patient survival. Bold font denotes modules showing significant splits at p-value <1e-3 (<1 false positive expected by chance); normal font denotes modules associated with survival at p-value <5e-3 (~1 false positive expected by chance). No. random samples, the number of random sample partitions (N = 526) exceeding the significance threshold for each tumor; No. random gene sets, number of random gene sets (N = 526) exceeding significance threshold; No. modules, total number of modules exceeding significance threshold; No. clusters, number of clusters of modules with overlapping genes (>25% overlap); selected top module, representative top module from a cluster comprising at least two modules with overlapping genes or similar annotation; top module annotation, representative annotation term associated with module (S2 Table);NA, not applicable. The modules reaching significance with Rectal tumors did not share gene overlap or annotation and were therefore considered “NA”.

A summary of this analysis is presented in Table 2. Consistent with Fig 1A, we observed significant survival differences in only a fraction of tumors (5/12 and 9/12 tumors at p-values <1e-3 and <5e-3, respectively). Kidney, melanoma, head and neck, bladder, and breast tumors were significantly associated with modules at the more stringent p-value (p <1e-3). Additional tumors (lung adenocarcinoma, glioblastoma, lung squamous and rectal tumors) were associated with specific modules at the less stringent p-value (p <5e-3). Survival of ovarian, uterine and colon patients was not significantly associated with any immunological theme or module in these tumors. With the random partitions, we generally found that more random gene set partitions than sample partitions yielded significant differences. Importantly, we did not find that most random gene sets were significantly associated with outcome as has been seen in other studies [24,25]. The higher number of positives with random gene partitions suggests that some genes possess prognostic value irrespective of the identities of other genes they are grouped with. We therefore considered using random sample partitions as a more reliable measure of the true null distribution for gene set comparisons. Irrespective of the method of random partitioning, numbers of modules yielding survival differences were higher than random sets for Kidney, Melanoma, Head and Neck and Bladder tumors. Consistent with Fig 1B, the patterns of modules associated with survival differences were complex, both within and between tumor types. Multiple immune processes were associated with survival with several tumors. For example, with melanoma, Interferon response modules gave the most significant differences, followed by Myeloid and T/NK cell modules [15]. Across tumor types, we observed several patterns of modules associated with survival. T/NK cell genes were most prominently associated with survival in melanoma, head and neck and bladder tumors, and less prominently with kidney, glioblastoma and lung squamous tumors. Myeloid cell genes were most prominently associated with survival in kidney, bladder, breast, and glioblastoma tumors. The Interferon response signature was predominantly associated with survival in melanoma, less so with kidney tumors. Together, these findings suggest that immune processes contributing to survival differences between tumors are complex, and unique for each tumor type.

The data summarized in Table 2 also show relatively few tumor types where T/NK cell modules were associated with significant survival differences (most significantly, kidney, melanoma, head and neck, and bladder). This finding is in contrast to other studies showing T cell infiltrates in numerous tumor types [10,11]. One potential explanation for the difference in our results is limited infiltration and expression of T/NK module genes in certain tumor types. To test this possibility, we identified four representative T/NK modules that tracked with survival differences in distinct tumor types (FOXP3.mod, PDCD1.mod, FLT3LG.mod and KLRB1.mod for bladder, head and neck, kidney and melanoma, respectively (Table 2)). We then plotted median expression levels of genes in these modules across all individual tumor samples (S2 Fig). This analysis showed relatively consistent levels of module gene expression across all tumor types (S2 Fig). The range of median module gene expression within tumor types was greater than between tumor types (S2 Fig and data not shown). These findings suggest that overall levels of T/NK cell infiltrates and T/NK gene expression across different tumors are more similar than levels between individuals with the same tumor type. Thus, variation in total expression level of module genes between tumor types does not explain the relatively low number of tumors where T/NK cell module genes were associated with significant survival differences.

### Distinct genetic alterations are associated with tumors having similar immune infiltrates but different prognoses

**Fig 2:**
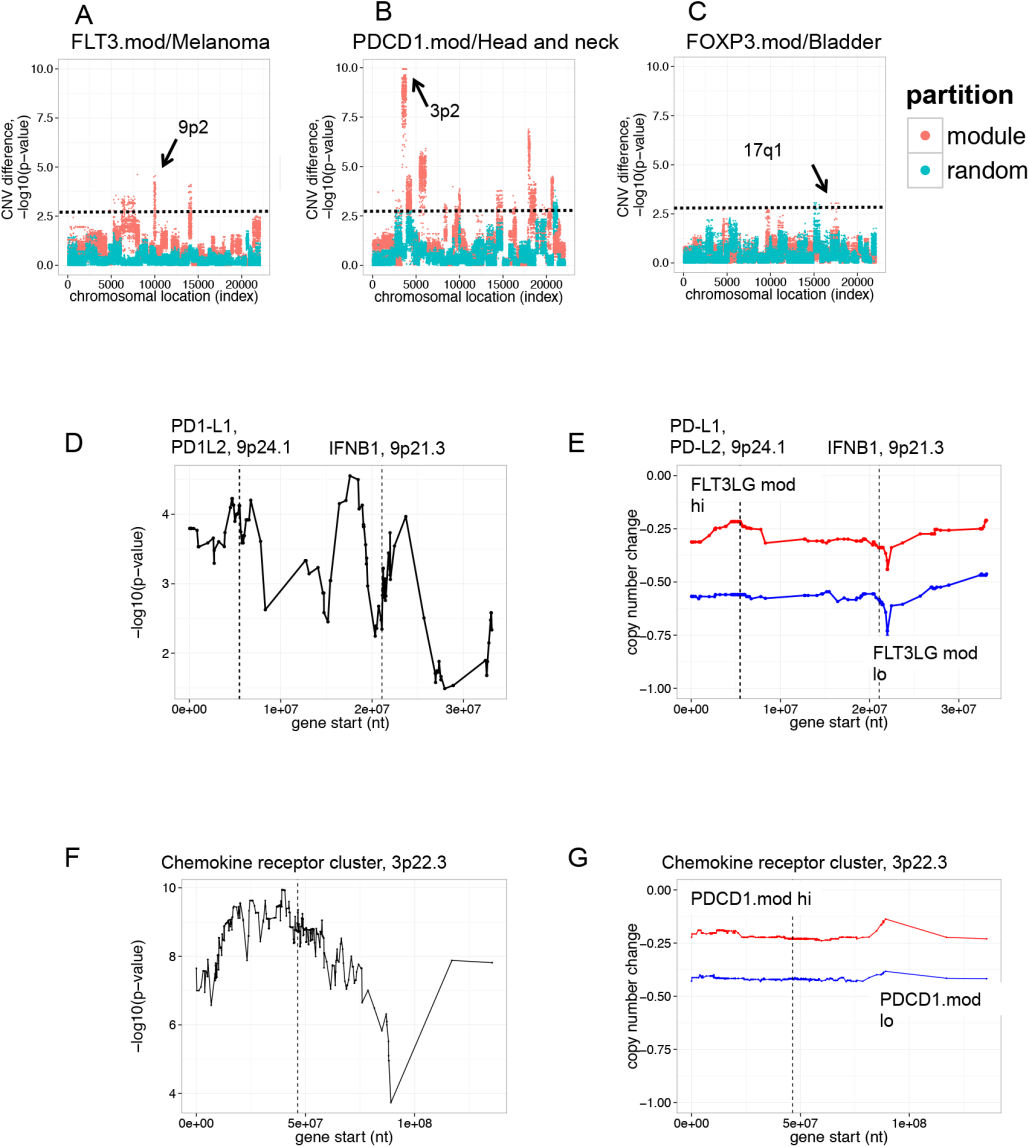
T cell infiltrates are associated with distinct genetic alterations in different tumor types. The significance (Wilcoxon p-value) of copy number differences between groups of T/NK module module hi and module lo tumors were determined for all genes in the genome. Shown are Manhattan plots for p-values (y axis) for all genes, arranged in ascending order (left to right) by chromosomal start position (x axis). Horizontal dashed lines denote significance cut-off of p-value = 1e-3. Arrows indicate regions of highest significance. Red, samples partitioned by module expression; teal, samples partitioned randomly. A) Melanomas partitioned by expression of T/NK cell module, FLD3LG.mod, showed significant copy number differences at chromosome 9p21. B) Head and neck tumors partitioned by expression of T cell module, PDCD1.mod, showed significant copy number differences at chromosome 3p22. C) Bladder tumors partitioned by expression of T/NK cell module, FOXP3.mod, showed significant copy number differences at chromosome 17q21. D) Significance of copy number differences between FLT3LG.mod hi and FLT3LG.mod lo tumors in melanoma across a large region of chromosome 9p2. Shown is an enlarged view of the significance of copy number difference in genes on chromosome 9p2. Dashed vertical lines indicate positions of IFNB1 and PD1-L1/PD2-L2 gene loci. For reference, the IFNB1 gene begins at chromosome 9 nucleotide 21,077,104, and PD-L2 at nucleotide 5,510,545. E) Copy number loss at 9p2 in FLT3LG.mod lo versus FLT3LG.mod hi melanomas. Shown is an enlarged view of median copy number change for genes on chromosome 9p2 in melanoma. Blue, FLT3LG.mod lo tumors (poor prognosis); Red, FLT3LG.mod hi tumors (good prognosis). F) Significance of copy number differences between PDCD1.mod lo and PDCD1.mod hi head and neck tumors across a region of chromosome 3p. Dashed vertical lines indicate the location of a cluster of chemokine receptor genes. G) Copy number loss at 3p2 in head and neck tumors. Blue, PDCD1.mod lo tumors (poor prognosis); Red, PDCD1.mod hi tumors (good prognosis).

Another factor that may influence how T/NK cell infiltrates influence patient survival is differences in microenvironment induced by genetic alterations in the tumor itself [26]. Our previous studies showed that poor prognosis melanomas with reduced ISG and immune gene levels were associated with specific copy number loss of the interferon gene cluster located at chromosome 9p21.3 [15]. It was important to determine whether immune gene copy number variation was associated with prognosis in tumors partitioned by module gene expression differences. We focused on tumors where T/NK cell gene expression most clearly predicted survival (melanoma, head and neck, and bladder tumors, Table 2). We used Wilcoxon p-values to determine significance of copy number differences for each gene in module hi and lo samples from melanoma, head and neck, and bladder tumors (S4 Table). For each tumor type, we plotted the significance of differences in copy number (p-values) for each gene versus its chromosomal start site (Fig 2A–2C). For comparison, we plotted significance of differences in copy number for randomly partitioned samples.

Melanoma samples with differing levels of expression of the T/NK cell module FLT3LG.mod showed highly significant copy number differences of multiple genes mapping to chromosome 9p2 when compared with randomly partitioned tumors (Fig 2A). This was consistent with our previous findings [15] showing copy number differences associated with loss of the interferon gene cluster in this region in a subset of melanomas [15]. Likewise, head and neck tumor samples differing in expression of the T/NK cell module PDCD1.mod showed highly significant copy number differences of genes at chromosome 3p2 (Fig 2B). FOXP3.mod hi and FOXP3.mod lo bladder carcinoma samples showed less pronounced copy number differences than the other tumor types, but still had an excess of significant genes at chromosome 17q1 when compared with randomly partitioned tumors (Fig 2C). Thus, at a group level, samples selected for differential expression of T cell genes exhibited distinct genetic alterations in different tumor types, and therefore comprised different molecular subtypes.

To explore in greater detail the extent of genetic alteration in melanoma samples selected for expression of FLT3LG.mod genes, we plotted p-values versus chromosomal location for all genes on chromosome 9p2 (Fig 2D). We found more extensive alterations in this region when compared with our previous studies on loss of the 9p21.3 locus in tumors expressing low ISG levels [15]. With FLT3LG.mod partitioned tumor samples, there were several peaks of significance on chromosome 9p2, comprising ~90 genes (Fig 2D, S4 Table). Examination of these genes revealed the interferon gene cluster at 9p21.3, as well as additional immune modulatory genes, including CD274 (PD1-L1) and PDCD1LG2 (PD1-L2), ligands for the T cell inhibitory receptor, PDCD1 (PD-1) (Fig 2D). Anti-PD1-L1 mAbs have shown clinical activity as therapeutics for multiple tumor types including melanoma [7,27].

Median copy number changes for each gene in the region were consistent with the p-value differences (Fig 2E). FLT3LG mod lo samples had reduced copy numbers for all genes in the region, compared with FLT3LG.mod hi samples, including PD-L1 and PD-L2 loci, in addition to IFN genes. In agreement with their reduced copy number, transcript levels for PD-L1 and PD-L2 were reduced in FLT3LG.mod lo tumors (S3 Fig).

**Fig 3:**
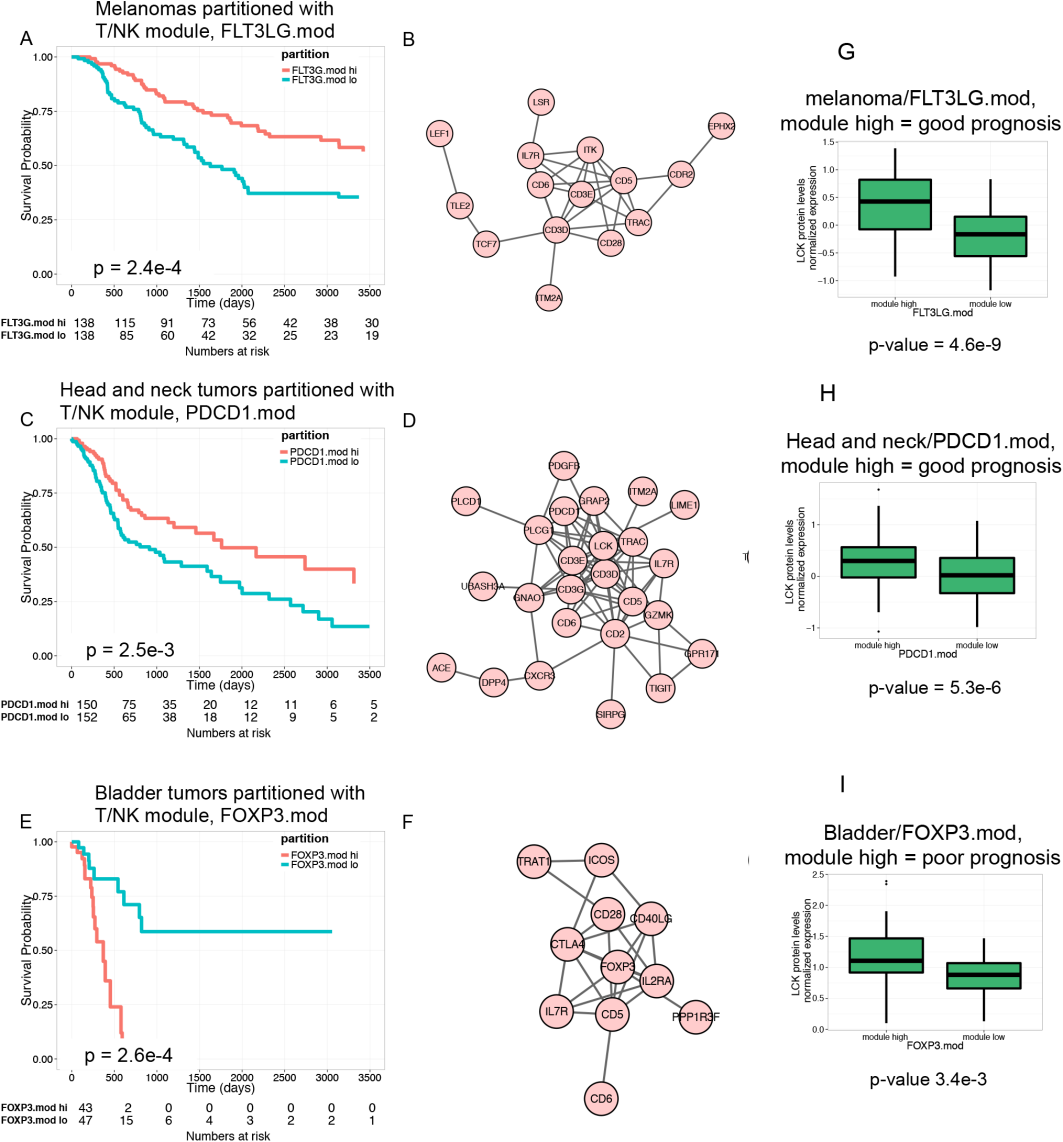
Expression of T/NK module genes may be oppositely associated with patient survival. A) Overexpression of FLT3LG.mod genes is positively associated with survival of melanoma patients. Shown is a KM plot depicting survival of melanoma patients stratified by expression of FL3LG.mod. FLT3LG mod hi, tumors having greater than median expression of module genes; FLT3LG.mod lo, tumors having less than or equal to than median expression of module. B). Network graph of FLT3LG.mod genes from a protein-protein interaction network shows interconnected T cell genes. C) Overexpression of PDCD1.mod genes is positively associated with survival of head and neck tumor patients. Shown is a KM plot depicting survival of head and neck tumor patients stratified by expression of PDCD1.mod. D). Network graph of PDCD1.mod genes shows interconnected T cell genes. E) Overexpression of FOXP3.mod genes is negatively associated with survival of bladder tumor patients. Shown is a KM plot depicting survival of bladder tumor patients stratified by expression of FOXP3.mod. F) Network graph of FOXP3.mod genes shows interconnected T cell genes, including Treg genes FOXP3, IL2RA and CTLA4. G-I) Tumors partitioned by levels of T/NK cell transcripts also show differences in levels of T cell protein, LCK. LCK expression was measured by RPPA. G) LCK protein levels in FLT3LG.mod hi versus FLT3LG.mod lo melanomas. H) LCK protein levels in PDCD1.mod hi versus PDCD1.mod lo head and neck tumors. I) LCK protein levels in FOXP3.mod hi versus FOXP3.mod lo bladder tumors.

Similar analyses for PDCD1.mod hi and lo tumors showed highly significant copy number differences between genes on chromosome 3p2 (Fig 2F). This analysis showed a peak of significance on chromosome 3p2, comprising ~500 genes (Fig 3F, S4 Table). Included in this region were members of the chemokine receptor gene cluster at 3p22.3 [28]. PDCD1.mod lo samples, when compared with PDCD1.mod hi samples, had reduced median copy numbers for all genes in the region, including a locus encoding multiple chemokine receptors (Fig 2G). In agreement with reduced their copy number, transcript levels for chemokine receptors CCR1, CCR2, CCR3, CCR5, CCR9, CCRL2, CXCR6 and XCR1 were reduced in PDCD1.mod lo tumors, relative to PDCD1.mod hi tumors (S4 Fig). Our studies are in alignment with previous studies showing that many cancers express a complex chemokine network that influences immune-cell infiltration, as well as tumor growth and invasion properties [29]. Taken together, these findings indicate that tumors stratified by expression of lymphocyte genes represent genetically distinct tumor subsets. Differences in gene expression accompanying chromosomal rearrangements may influence levels of immune modulatory molecules in the tumor microenvironment and contribute to better or worse patient survival prognosis.

### Similar immune processes may affect patient survival either positively or negatively depending on tumor type

Data shown in Table 2 indicate that T/NK module gene expression is associated with survival differences in different tumors. However, these data do not provide insight into whether high or low module gene expression is associated with better prognosis. To test these associations in different tumor types, we selected three T/NK cell modules (FLT3LG.mod, PDCD1.mod and FOXP3.mod that were associated with patient survival (in melanoma, head and neck and bladder tumors, respectively). We then plotted Kaplan-Meier (KM) curves for survival of T/NK module hi and module lo tumors. (Fig 3).

Consistent with our previous studies [15], FLT3LG.mod hi melanoma patients had better prognosis than patients with FLT3LG.mod lo tumors (Fig 3A). To better understand the nature of the genes associated with this survival difference, we projected FLT3LG.mod genes onto a protein-protein interaction (PPI) network graph (Fig 3B). These genes formed an interconnected network of genes expressed in a wide variety of T cell types (Fig 3B). Likewise, head and neck tumors with elevated levels of PDCD1.mod genes were associated with better patient survival (Fig 3C). Projection of PDCD1.mod genes onto a PPI also revealed an interconnected network of pan-T cell genes (Fig 3D), overlapping with the FLT3.mod network (Fig 3B). In contrast, FOXP3.mod hi bladder tumors were associated with worse patient survival (Fig 3E). Projection of FOXP3.mod genes on to a PPI revealed an interconnected network that contained several genes in common with FLT3LG.mod and PDCD1.mod networks (CD5, CD6, CD28, IL7R, etc., Fig 2F). In addition, however, the FOXP3.mod network contained several genes important for the development and function of regulatory T cells (Treg): FOXP3 (forkhead box P3); IL2RA (IL2 receptor alpha subunit); and CTLA-4 (Cytotoxic T lymphocyte-asociated-4). These findings demonstrate that high levels of Treg genes, relative to genes found in other types of T cells, are associated with poor patient prognosis in bladder carcinoma. Tregs would be expected to reduce inflammation and potentially inactivate cytoxic T cells, preventing immune-mediated tumor destruction. The presence of Tregs within bladder tumors has been associated with poor patient prognosis [30]. In the other tumor types having reduced ratios of Treg genes to other T cell genes (i.e., melanoma and head and neck tumors), the presence of a T/NK signature is associated with improved prognosis. Taken together, our results indicate that elevated levels of T cell genes may be associated with either better or worse patient survival in different tumor types, depending upon the type of T cell infiltrate.

We also observed either better or worse patient survival, depending on tumor type, in patients having tumors with high levels of Myeloid cell gene expression. For example, neutrophil module CTSG.mod hi kidney tumors were associated with poor patient survival (S5A Fig). CTSG (Cathepsin G) is associated with a network of genes (S5B Fig) involved in neutrophil function, including: ELANE (neutrophil elastase); AZU1 (Azurocidin 1); DEFA4 (defensin, alpha 4); LTF (lactotransferrin); and MPO (myeloperoxidase). However, in breast tumors, DEFA3.mod hi tumors were associated with better patient survival (S5C Fig). DEFA3.mod genes form an interconnected network involving (S5C Fig) many of the same genes as in CTSG.mod (S5B Fig).

To provide evidence for the relevance of our findings at the protein level, we analyzed tumor protein expression by RPPA. Proteins measured by the TCGA group included the kinase LCK; LCK is an integral component of T cell receptor signaling, a T cell marker, and a node in the sub-network shown in Fig 2D. Melanoma, head and neck, and bladder tumors expressing high levels of FLT3LG.mod, PDCD1.mod and FOXP3.mod transcripts, respectively, also expressed significantly higher levels of LCK protein (Fig 2E–2G). This finding demonstrates that all three module hi tumor sets contain higher levels of T cell infiltrate than their corresponding module lo sets. The difference in prognosis between melanoma and head and neck versus bladder module hi tumors therefore likely reflects differences in functional properties of the T cells in their respective infiltrates. Treg cells in bladder tumors may contribute to an immunosuppressive state that leads to tumor escape rather than elimination by the immune system [31].

## Discussion

We have used a non-biased and data driven systems approach to identify transcript modules that represent specific immune cells or processes associated with patient survival in a dozen different tumor types. Numerous studies have shown that infiltrates of selected immune cell types are associated with survival of patients with individual tumor types (reviewed in [10,11]). However, our study is unique in our association of many immune cell processes with survival of patients across multiple tumor types.

Our studies show that expression of immune genes is associated with patient survival across some, but not all tumor types. Using a stringent cut-off to minimize false positive detections, we identified immune gene modules whose expression was associated with patient survival only in kidney, melanoma, head and neck, bladder, and breast tumors. In contrast, we did not find immune gene module expression associated with patient survival in 4/12 tumor types tested (rectal, colon, uterine and ovarian tumors). We could not attribute these differences to technical factors like sample size, or to survival characteristics of the particular cohorts used. Our data therefore suggest that specific tumor types may be more or less susceptible to control by the immune system.

Anecdotal evidence has long supported the notion that certain tumor types are inherently more responsive to immune therapy (i.e., “immunogenic”) than others. For instance, immunotherapy is most often effective against melanomas [1], which may regress spontaneously [32] or in response to therapy [33] concomitant with autoimmune symptoms. Moreover, current FDA approved “active” immunotherapies for solid tumors [22] are limited to only a few tumor types (e.g., melanoma, bladder, kidney, etc.). Our data shows that these “immunogenic” tumors tend to be those where we most clearly identified immune gene expression associated with patient prognosis. This suggests that tumors with more active basal levels of immune processes are more amenable to immune-based therapy.

We observed distinct patterns of modules associated with survival in different tumors. Survival of kidney and breast tumor patients was most related to levels of innate cells (neutrophils and myeloid cells), whereas for melanoma, head and neck, and bladder tumors, survival was most related to levels of adaptive immune response genes (T/NK cells). Surprisingly, we identified predominant T cell effects on survival in only a few tumor types (most significantly melanoma, head and neck carcinoma, and bladder tumors). This contrasts with the variety of tumor types where T cell infiltrates have been associated with favorable prognosis in the literature [10,11]. This difference may result from the patient cohorts studied, or from methodologies used to assess significance. Importantly, since we relied on a population measure for gene expression (median), our results do not rule out roles for T cells in regulating survival of small subsets of the tumor types that scored negatively in our tests. Also, our methods are unlikely to detect differences in intra-tumoral localization of immune cells which can affect patient survival [8]. Nonetheless, our side-by-side testing strongly suggests relative differences in the extent to which T cells are associated with patient survival across tumor types.

Because of the unique scope and clinical characteristics of the TCGA tumor sets, they are an invaluable resource for molecular characterization of tumors [13,34,35]. For characterization of a particular tumor type, it is important to consider how TCGA data are replicated in independent test sets. We previously confirmed in an independent study that ISG and T cell gene sets were associated with melanoma patients’ survival, just as they were with TCGA samples [15]. For the present studies, we have been unable to locate profile sets of adequate size or comparable clinical characteristics to validate our findings with bladder and kidney tumors. However, in agreement with our results, previous studies have found elevated neutrophil levels in later stage renal cell carcinomas [36]. Poor prognosis has also been associated with a high pre-treatment neutrophil-lymphocyte ratio in kidney tumors [37]. Also in agreement with our results, FOXP3+ infiltrates have been observed in bladder carcinomas [30], although these have not yet been associated with poor prognosis.

We have shown that tumor subgroups selected on the basis of immune cell gene expression often show distinct genetic alterations. Long-range chromosomal alterations may disrupt production of entire families of immune modulators, which frequently are encoded in gene clusters. Chromosomal instability was demonstrated to be a mechanism of modulating local cytokine expression in colorectal tumors [26]. Likewise, our previous studies showed that reduced expression of ISGs in melanomas was associated with poor patient prognosis, and with copy number loss of the interferon gene cluster located at chromosome 9p21.3 [15]. In the present study, we show that a subset of melanoma patients associated with reduced T cell infiltrate may lose chromosomal sequences at 9p2. This new locus is many megabases away from the interferon gene cluster we previously linked to reduced interferon gene expression and poor prognosis. These additional sequences encode the PD-L1 and PD-L2 immune modulatory genes, which negatively regulate T cell proliferation and are targets of immunotherapeutic mAbs [7,27].

We also show that a cluster of chemokine receptor genes at chromosome 3p2 is lost in head and neck tumor patients selected for reduced expression of PDCD1-linked T cell genes. Cancer cells oftentimes subvert the chemokine system, such that chemokines and their receptors become important regulators of cell movement into and out of the tumor microenvironment [38]. Several members of the chemokine receptor gene cluster at 3p22.3 (CCR1, CCR3. CCR5, CCRL2, XCR1) are receptors for T cell chemokines, including CCL5 (RANTES), XCL1 (lymphotactin) and RARRES2 (chemerin) [28]. This suggests a possible relationship between T cells expressing a network of PDCD1 associated genes and the chemokine receptor cluster at 3p2 in head and neck tumors. Overall, therefore, our findings contribute to emerging evidence that genomic rearrangements within tumors can modulate the tumor microenvironment and influence anti-tumor immunity.

Our findings suggest new potential biomarkers for patient selection that could improve the successful use of cancer immunotherapies. PD-L1 and PD-L2 expression were reduced in tumors having copy number loss at chromosome 9q2. PD-L1 expression was the factor most closely correlated with therapeutic response to anti-PDCD1 monoclonal antibody (mAb) [12]. Other studies have suggested that anti-PD-L1 immunotherapy is most effective in patients where existing PD-L1/PDCD1 interactions are available for reversal by anti-PD-L1 mAbs [7]. Our findings therefore suggest that melanoma patients with high copy number at 9q2 may be better candidates for anti-PD-L1 therapy than patients with reduced copy number and expression of PD-L1 and PD-L2. Likewise, our demonstration of improved survival in a subset of head and neck tumor patients with elevated levels of PDCD1-linked gene expression and alterations at chromosome 3p2 suggests that this is a population of head and neck tumor patients who may benefit from anti-PDCD1/PD-L1 therapy.

Our results also suggest that immunosuppressive environments involving either T/NK or Myeloid cells, depending on tumor context, may influence the ability of the immune system to control tumor growth. We showed that high levels of a gene network including the T regulatory (Treg) gene FOXP3 are associated with poor survival of bladder tumor patients. Likewise, we showed that high levels of a neutrophil gene network including CTSG (Cathepsin G, a component of the azurophilic granules of neutrophils) are associated with poor survival of kidney tumor patients. Both Tregs [39] and azurophilic granules [40] have previously been associated with immune suppressive function in tumors. Our findings, therefore, support the seemingly contradictory notion that the immune system may have both host-protective and tumor-promoting effects on developing tumors [31]. Moreover, our findings suggest a framework for molecular categorization of these effects, which will enable finer control of immunotherapy.

Finally, our results suggest that T regulatory cells (Tregs) might be important therapeutic targets in certain tumor types. Animal studies have clearly shown the inhibitory effects of Tregs on anti-tumor immunity [39]. This suggests that strategies to deplete tumor-associated Tregs might enhance anti-tumor immunity and cause tumor shrinkage in humans. However, efforts to therapeutically target Tregs have met with mixed success [41]. Indeed, Tregs are not currently portrayed as a major target for cancer immunotherapy [1]. Here we show evidence for significant negative association of FOXP3-linked genes and patient survival in only 1/12 tumors tested (bladder tumors). This suggests that identifying suitable patients and tumor types may be a major limitation for Treg-directed therapies. Our finding that FOXP3-linked genes are associated with poor prognosis in a subset of bladder tumor patients suggests a specific patient subset predicted to benefit from therapeutic reduction of FOXP3+ Tregs. Taken together, these findings suggest that personalized approaches towards selection of specific patient subsets within and between tumor types will aid in optimizing cancer immunotherapies.

## Experimental Procedures

### The Cancer Genome Atlas (TCGA) Data

TCGA data designated as available without restrictions were obtained from public repositories. Data and sample annotation for the SKCM (melanoma) set were obtained from the Broad Institute GDAC Firehose (https://confluence.broadinstitute.org/display/GDAC/Home). RNAseq profiles and sample annotation from the PANCANCER12 set comprising RNAseq profiles from 11 different tumor types were obtained from Synapse (www.synapse.org). Tumors were stratified into groups by expression of immune molecular module genes [15]. We used median expression of genes in each module to divide each tumor data set into two groups, one with higher and one with lower than median gene expression. Survival analysis was performed on the two groups using the date of last visit for each subject.

RNAseq profiles each comprised ~20 million reads normalized by the RSEM procedure [42]. Expression values for RNAseq data are reported as log2 (Reads per million (RPM)+1). GISTIC2 copy number variation (CNV) and Reverse phase protein array data (RPPA) data were obtained from the GDAC Firehose as level 4 Standard Data.

### Derivation of transcript modules

Immune molecule modules were derived as described previously [15], but using increased sample numbers and additional marker genes. Modules were derived from coexpression matrices between levels of marker genes and all other genes across a dataset of RNAseq profiles. Marker genes included all known CD antigens and cytokines, as well as transcription factors found in human hematopoietic cells [43]. The dataset comprised profiles from 336 samples of whole blood and/or purified cells (CD4+ T cells, CD8+ T cells, NK cells, B cells, neutrophils, and monocytes) from healthy controls and individuals with autoimmune or infectious diseases. We calculated Pearson correlation coefficients between levels of marker gene transcripts and levels of transcripts matching 50,045 ENSEMBL gene models, across all samples. We arbitrarily chose the top 100 transcripts most positively correlated with each marker (Pearson correlation coefficients, 0.228–1.00, median = 0.831) and designated these gene sets as immune molecular modules (S1 Table). In general, correlation coefficients were weaker for transcripts negatively correlated with markers; these transcripts were not considered further. Many transcripts in immune molecular modules also were also positively correlated with markers across the combined melanoma/PANCANCER data sets (top quartile and median correlation coefficients, 0.14 and 0.036, one-tailed p-values = 0.0160 and <1e-8, respectively). The 526 modules comprised ~9,070 named genes. For random gene set controls, we randomly selected N = 526 sets of 100 genes with substitution.

### Clustering and annotation of transcript modules

Our procedure for creating transcript modules results in groups (clusters) of modules with many overlapping genes [15]. This is to be expected because of the highly similar distributions of marker genes (e.g., CD19 and CD20 for B cells). To systematically identify clusters of related immune molecular modules, we calculated the fraction of pairwise gene overlap between all 526 different immune molecule modules. We then clustered the resulting overlap table using the *hclust* function in R (Ward D method). We determined the optimal number of clusters (N~18) using the *mclust* package in R. The grouping of immune molecular modules into clusters is presented in S2 Table.

To associate functional terms with immune modules, we compared them with gene sets in reference databases, including: current MSigDB gene sets (http://www.broadinstitute.org/gsea/downloads.jsp); ‎gene sets selective for different hematopoietic cells [17]; and blood transcriptional modules (BTM) [18]. We determined the significance of gene overlap for all pairwise combinations of modules and annotated gene sets using the hypergeometric distribution. We corrected p-values for multiple testing using the Benjamini–Hochberg procedure in R, and considered a false discovery rate (FDR <0.05 as a significance cut-off for annotation terms. The top (most significant) annotation terms associated with individual immune molecular modules from each of three reference databases are presented in S2 Table. Each cluster of immune molecular modules was manually assigned a Summary annotation term consistent with the terms associated each module in the cluster (S2 Table).

### Analysis procedures

For network viewing, we projected gene lists onto the STRING 9.1 [44] Network of Known and Predicted Protein-Protein Interactions (http://string-db.org/). Nodes having ≥2 edges were then exported into Cytoscape [45] (http://www.cytoscape.org/) for manipulation and visualization. Network functional enrichment with GO Biological process terms was verified with STRING 9.1 or GeneMANIA [46] (http://www.genemania.org). We considered a False Discovery Rate (FDR) of <0.05 as significant for term enrichment.

Other analyses were performed using the R language and core packages [47], as well as additional packages: *ggplot2* [48]; *reshape2* [49]; and *survival* [19,50]. Concordance index values were calculated using the *survival* package. The *ggkm* function in R was used for plotting enhanced KM plots [51]

### Type I error correction and statistical significance

For survival curve difference (*survdiff)* p-values, we used permutation testing to correct for type I error and to estimate statistical significance directly from the data [15]. From 1,000 random partitions of samples from each tumor into equally sized groups, we observed an average of ~1 partition for each tumor type that predicted patient survival at *survdiff* p-values <1e-3, and ~4 modules at p-values <5e-3. Our cut-off p-values of 1e-3 and 5e-3 corresponded to median Benjamini-Hochberg false discovery rates (FDR) across different tumor types of 2e-3 and 8e-3, respectively.

## Supporting Information

S1 Fig Expression of immune module genes in tumors predicts patient survival in some but not all tumor types. Tumors were partitioned into equal sized groups by median gene expression of immune molecular modules (S3 Table). Shown is a boxplot representation of–log10 p-values for survival curve differences between module hi and module lo subsets for all tumor types partitioned by each module. For comparison, p-values for random partitions are shown. The horizontal line indicates a p-value = 5e-3. At this p-value, we expected ~2 partitions to result in significant survival curve differences by chance. Modules, significance of survival curve differences after partitioning by modules (N = 526); random, significance of survival curve differences after partitioning at random (N = 526 permutations).
(TIF)

S2 Fig Expression of T/NK gene modules is similar across different tumor types. Shown are plots of median expression of genes in T/NK modules FOXP3.mod, PDCD1.mod, FLT3LG.mod and KLRB1.mod for all tumor samples. These modules yielded significant survival curve differences in bladder, head and neck, melanoma and kidney tumors, respectively. x axis, arbitrary sample number; y axis, median module gene expression (log2 (RPM+1)); horizontal dotted lines demark boundaries of samples from different tumor types.
(TIF)

S3 Fig Different molecular subtypes of melanoma selectively express PD-1 ligands. Reduced expression of PD1 ligands, PD1-L1 and PD1-L2 in FLT3LG.mod lo versus FLT3LG.mod hi melanoma tumors. Shown are log2 values of normalized transcript counts (RPM +1) for the indicated genes. Differences between both module sets were significant (p-values <1.2e-9, Wilcoxon test).
(TIF)

S4 Fig Different molecular subtypes of head and neck tumors selectively express chemokine receptors. Reduced expression of chemokine receptor genes in PDCD1.mod lo versus PDCD1.mod hi tumors in head and neck tumors. Shown are log2 values of normalized transcript counts (RPM +1) for the indicated genes. All differences between module sets were significant (p-values <2.2e-7, Wilcoxon test).
(TIF)

S5 Fig Expression of Neutrophil module transcripts is oppositely associated with patient survival in different tumor types. A) KM plot showing poor survival of neutrophil module CTSG.mod hi kidney tumor patients. B). Protein-protein interaction network [44] of GTSG.mod genes shows an interconnected network of neutrophil genes. C) KM plot showing enhanced survival of neutrophil module DEFA3.mod hi breast tumor patients. D) Protein-protein interaction network of DEFA3.mod genes shows an interconnected network of neutrophil genes.
(TIF)

S1 Table Genes in immune molecular modules. Sheet 1, shown are the top 100 genes best correlated in expression levels with marker genes across row 1 (Experimental Procedures). Sheet 2, random sets of 100 genes.
(XLSX)

S2 Table Clustering and functional annotation of immune molecular modules. Shown are immune molecular modules from S1 Table organized as clusters by the extent of gene overlap between clusters. Also shown are Msig, hematopoietic cell and BTM terms most highly enriched in each module, as well as a manually assigned Summary annotation term. NS, not significant (FDR >0.05); Not determined, summary annotation not clearly identified. See Experimental Procedures for details.
(TXT)

S3 Table Prediction of patient prognosis by immune molecular modules. Immune molecular modules were scored for their ability to predict survival of different tumor types. Full_set, sheet showing p-values for survival curve differences for all tumor types partitioned by all modules; <5_year_survival, sheet showing survival curve differences for the subset of data showing < 5 years survival; Concordance, sheet showing Concordance index, a measure of prognostic value of signature scores [20,21] and p-values for survival curve differences for all tumor types partitioned by all modules.
(XLSX)

S4 Table Copy number differences between T cell module hi and T cell module lo tumors. Shown are median copy numbers for each gene in FLT3LG.mod, PDCD1.mod and FOXP3.mod hi and lo samples, in melanoma, head and neck carcinoma and bladder carcinoma tumors, respectively. Also shown are Wilcoxon p-values for differences in gene copy number between the groups. Since adjacent genes subject to copy number variation at the chromosomal scale were unlikely to vary independently, we did not use multiple testing corrections for this analysis. Instead, we used random partitions of the data to assess false positives (Fig 2A–2C).
(XLS)
