# Supplementary figures and images for: The Relationship of Immune Cell Signatures to Patient Survival Varies within and between Tumor Types
Source: PLoS One. 2015 Sep 23;10(9):e0138726. doi: 10.1371/journal.pone.0138726 (PMC4580625; doi:10.1371/journal.pone.0138726)

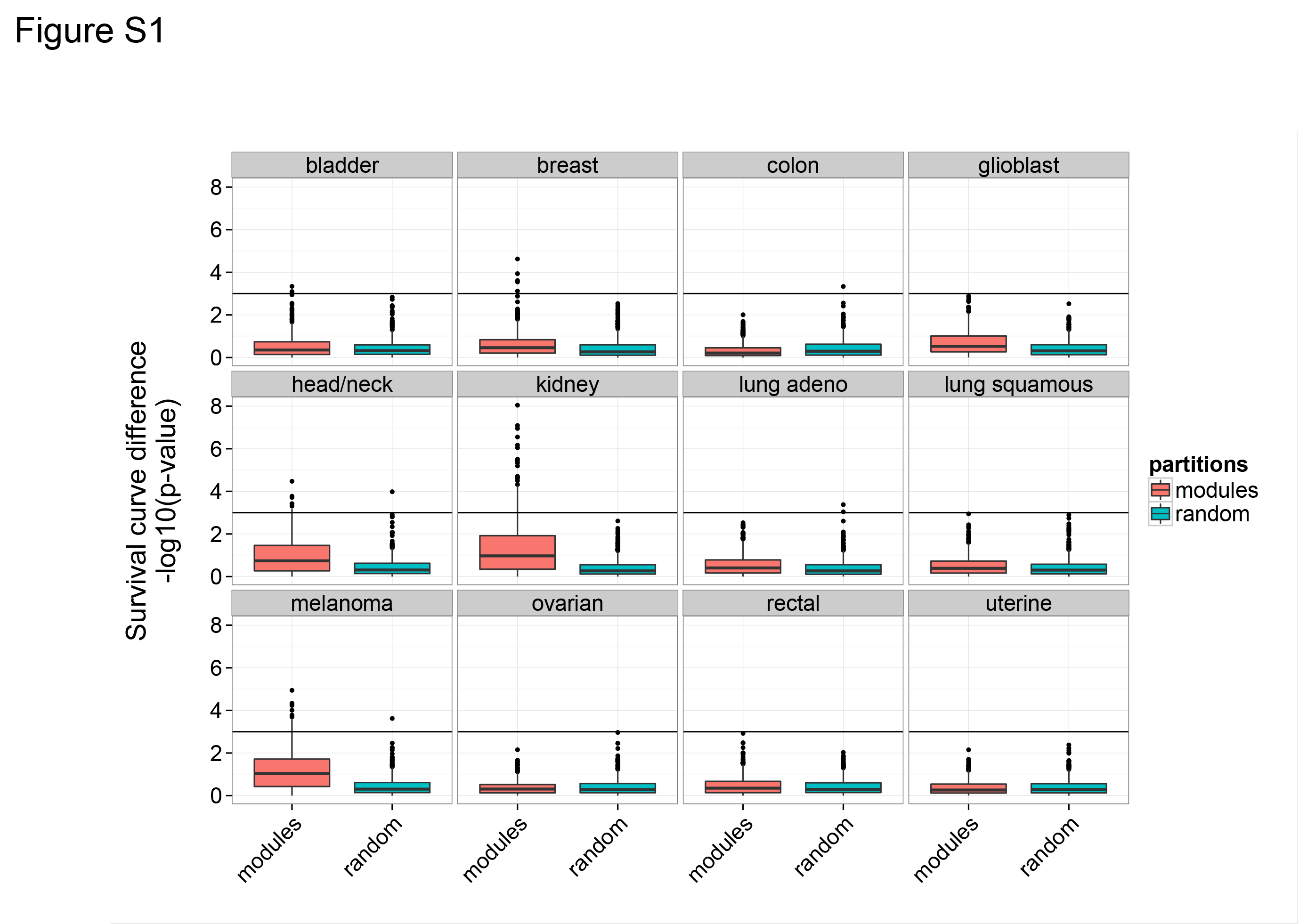

Supplement: S1 Fig — Tumors were partitioned into equal sized groups by median gene expression of immune molecular modules (S3 Table). Shown is a boxplot representation of–log10 p-values for survival curve differences between module hi and module lo subsets for all tumor types partitioned by each module. For comparison, p-values for random partitions are shown. The horizontal line indicates a p-value = 5e-3. At this p-value, we expected ~2 partitions to result in significant survival curve differences by chance. Modules, significance of survival curve differences after partitioning by modules (N = 526); random, significance of survival curve differences after partitioning at random (N = 526 permutations). (TIF) [file pone.0138726.s001.tif]

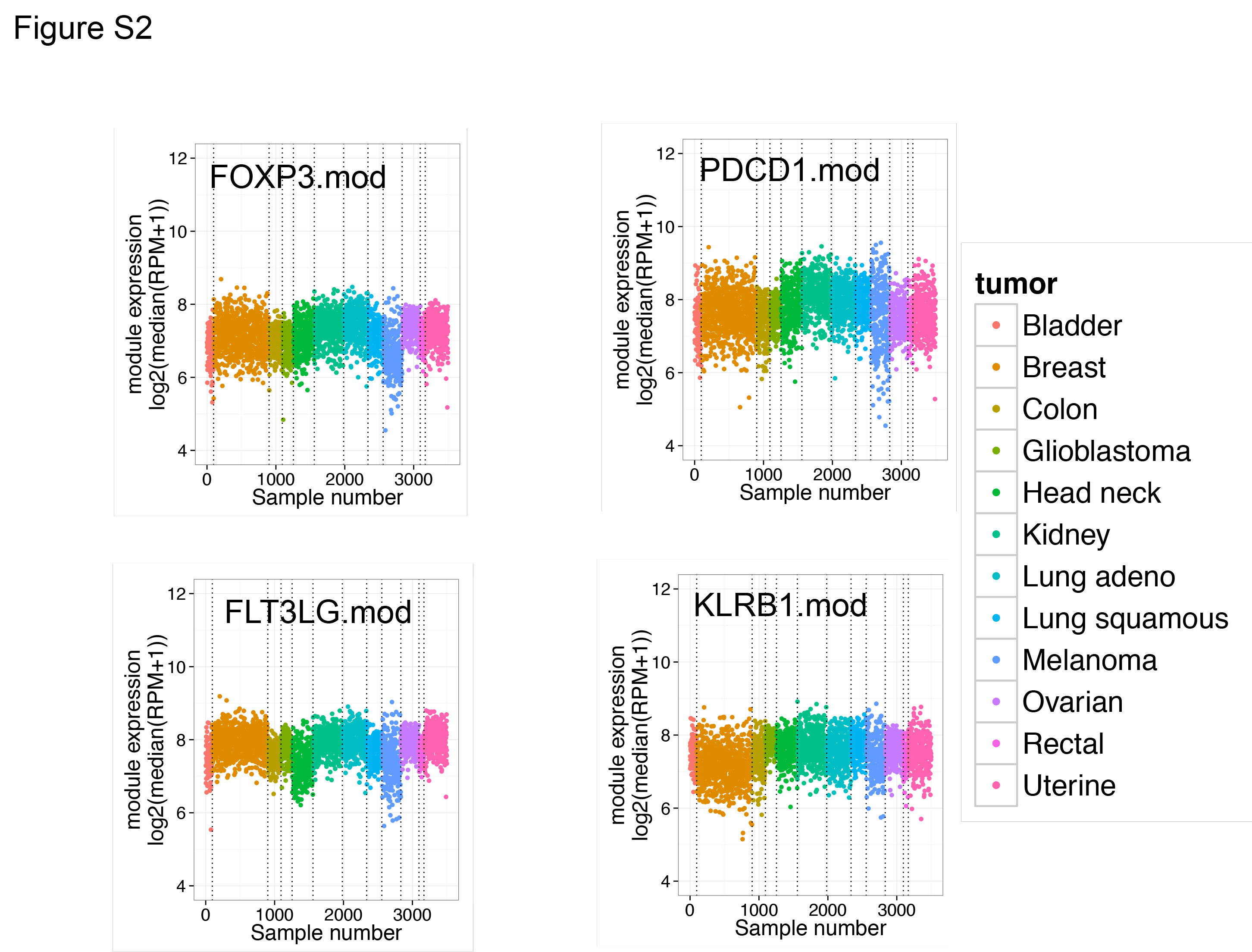

Supplement: S2 Fig — Shown are plots of median expression of genes in T/NK modules FOXP3.mod, PDCD1.mod, FLT3LG.mod and KLRB1.mod for all tumor samples. These modules yielded significant survival curve differences in bladder, head and neck, melanoma and kidney tumors, respectively. x axis, arbitrary sample number; y axis, median module gene expression (log2 (RPM+1)); horizontal dotted lines demark boundaries of samples from different tumor types. (TIF) [file pone.0138726.s002.tif]

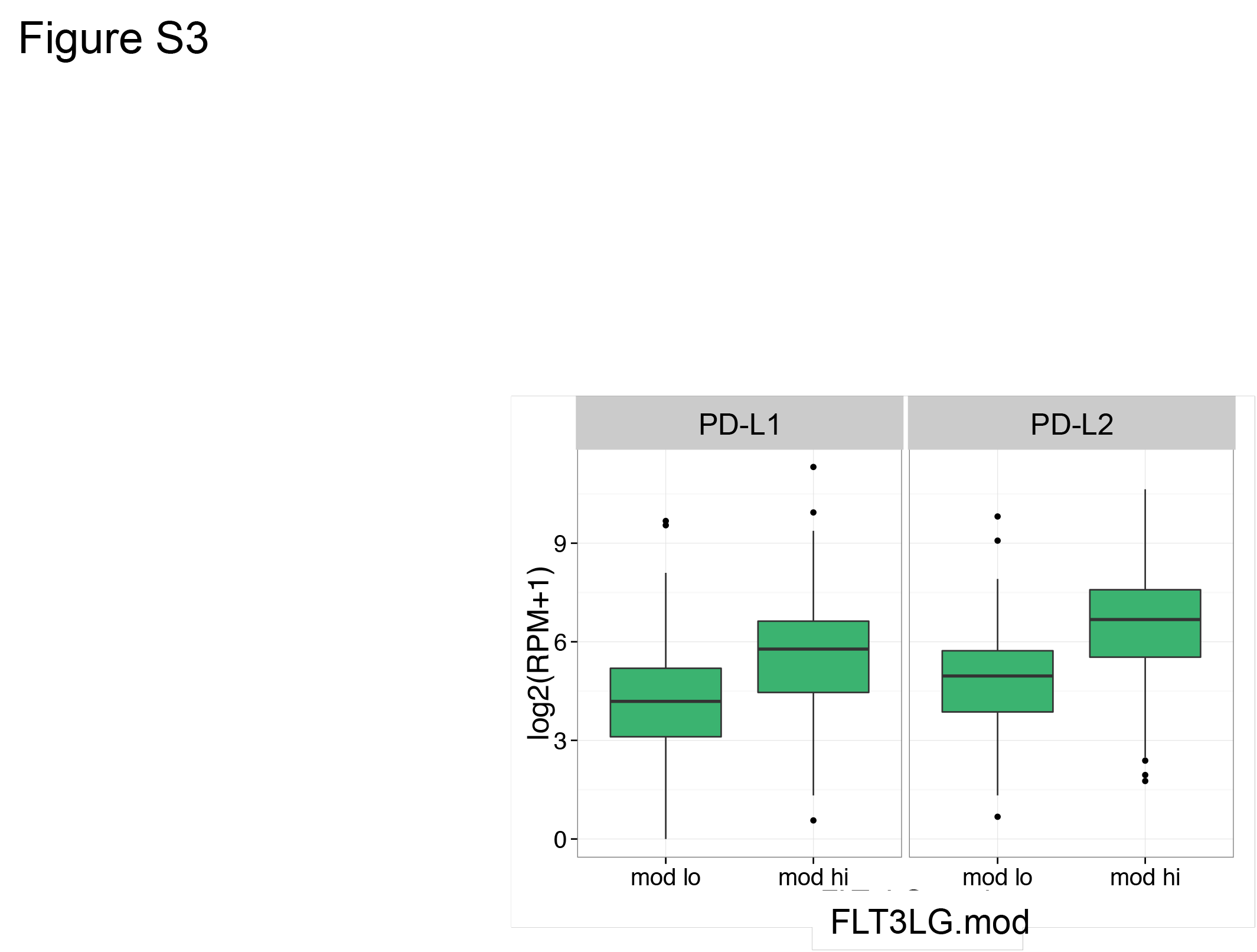

Supplement: S3 Fig — Reduced expression of PD1 ligands, PD1-L1 and PD1-L2 in FLT3LG.mod lo versus FLT3LG.mod hi melanoma tumors. Shown are log2 values of normalized transcript counts (RPM +1) for the indicated genes. Differences between both module sets were significant (p-values <1.2e-9, Wilcoxon test). (TIF) [file pone.0138726.s003.tif]

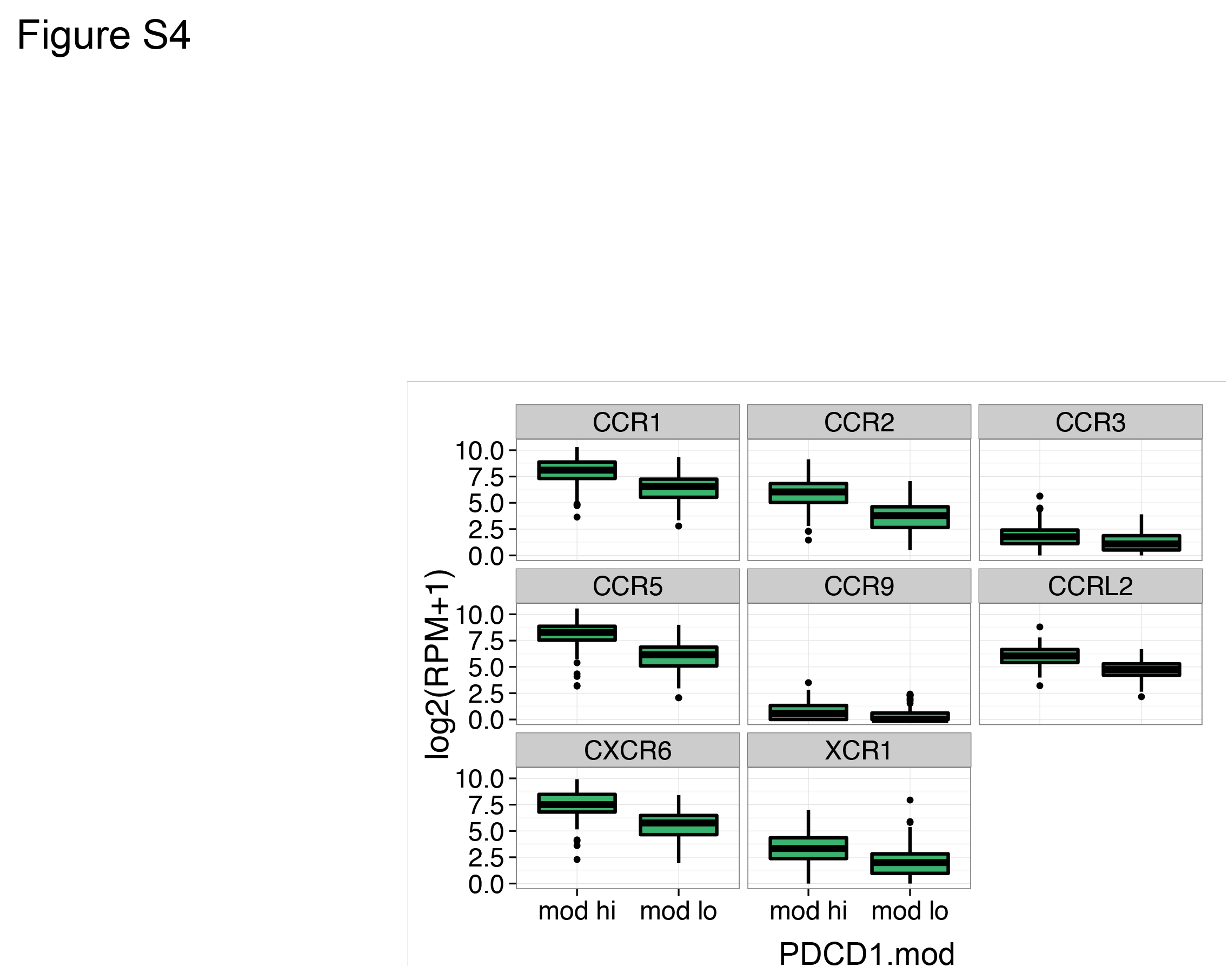

Supplement: S4 Fig — Reduced expression of chemokine receptor genes in PDCD1.mod lo versus PDCD1.mod hi tumors in head and neck tumors. Shown are log2 values of normalized transcript counts (RPM +1) for the indicated genes. All differences between module sets were significant (p-values <2.2e-7, Wilcoxon test). (TIF) [file pone.0138726.s004.tif]

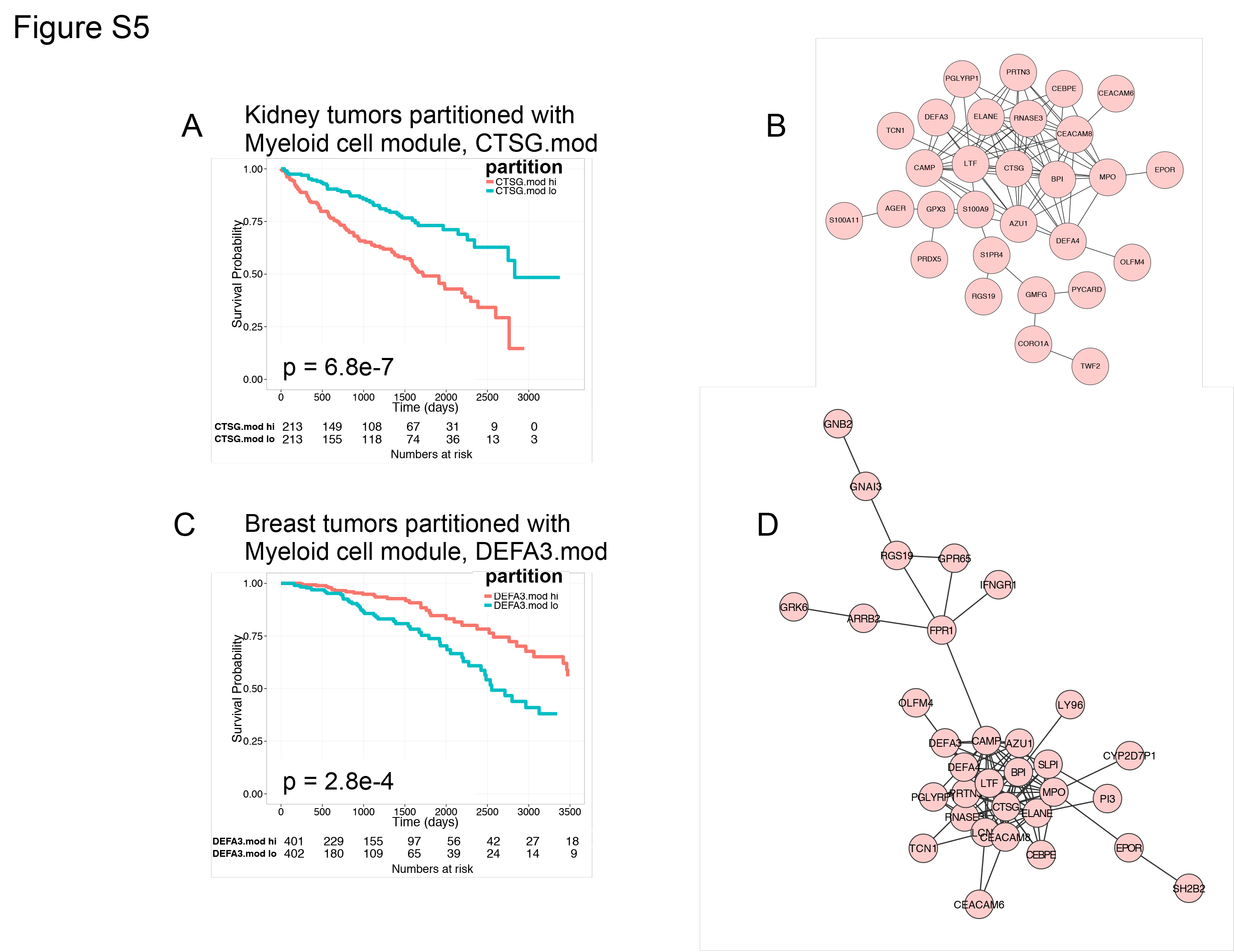

Supplement: S5 Fig — A) KM plot showing poor survival of neutrophil module CTSG.mod hi kidney tumor patients. B). Protein-protein interaction network [44] of GTSG.mod genes shows an interconnected network of neutrophil genes. C) KM plot showing enhanced survival of neutrophil module DEFA3.mod hi breast tumor patients. D) Protein-protein interaction network of DEFA3.mod genes shows an interconnected network of neutrophil genes. (TIF) [file pone.0138726.s005.tif]
